# Absence of Host-Specific Hemotropic Mycoplasmas in Horses and Donkeys from Croatia: First Systematic Survey in Southeastern Europe

**DOI:** 10.3390/ani16020263

**Published:** 2026-01-15

**Authors:** Nika Konstantinović, Jelena Gotić, Mirjana Baban, Goran Csik, Ema Listeš, Ema Gagović, Daria Jurković Žilić, Ivan Arežina, Gordan Šubara, Franka Emilija Čulina, Nika Delić, Dora Višal, Zlatko Zvonar, Relja Beck, Antun Kostelić

**Affiliations:** 1 Faculty of Veterinary Medicine, University of Zagreb, 10 000 Zagreb, Croatia; nkonstantinovic@vef.unizg.hr (N.K.); jselanec@vef.hr (J.G.); fculina@vef.hr (F.E.Č.); ndelic@vef.hr (N.D.); dvisal@vef.hr (D.V.); 2 Faculty of Agrobiotechnical Sciences Osijek, J.J. Strossmayer University, 31 000 Osijek, Croatia; mbaban@fazos.hr; 3 Veterinary Clinic Equivet d.o.o., 10 000 Zagreb, Croatia; goran.csik@gmail.com; 4 Linkomed Veterina d.o.o., 21 230 Sinj, Croatia; emalistes19@gmail.com; 5 Croatian Veterinary Institute, 10 000 Zagreb, Croatia; gagovic@veinst.hr (E.G.); jurkovic@veinst.hr (D.J.Ž.); 6 Veterinary Clinic Gospić, 53 000 Gospić, Croatia; ivanarezina1@gmail.com; 7 Agency for Rural Development of Istria (AZRRI), 52 000 Pazin, Croatia; gsubara@azrri.hr; 8 Miagro Veterina d.o.o., 31500 Lipine, 10 000 Zagreb, Croatia; opgzvonar@gmail.com; 9 Faculty of Agriculture, University of Zagreb, 10 000 Zagreb, Croatia; akostelic@agr.hr

**Keywords:** *Mycoplasma wenyonii*, horses, donkeys, Croatia, molecular, prevalence

## Abstract

Hemotropic mycoplasmas (hemoplasmas) are uncultivable, cell wall-less bacteria that parasitizeon the surface of red blood cells of mammals, potentially causing anemia and other systemic signs. While widely distributed among domestic and wild animals, their occurrence in equids remains poorly understood, and no species has been identified as host-specific to horses or donkeys. This study presents the first systematic survey of hemoplasmas in equids from southeastern Europe and only the second molecularly confirmed case in horses in Europe. A total of 843 equids (817 horses and 26 donkeys) from different regions of Croatia, representing various ages, uses, and husbandry systems, were screened for hemoplasmas by PCR targeting the 16S rRNA gene. Only one horse tested positive, identified as *Mycoplasma wenyonii*, a hemoplasma typically associated with cattle. The estimated prevalence was 0.12% (95% CI: 0.003–0.68%). No donkeys were infected. The extremely low prevalence observed here—the lowest reported in any study detecting hemoplasma-positive horses—supports the hypothesis that equids do not harbor host-specific hemoplasma species and may only sporadically acquire infections from other hosts via spillover. This finding underscores the apparent absence of persistent hemoplasma lineages adapted to equids and highlights the need for further research on their epidemiology, host specificity, and transmission dynamics.

## Simple Summary

We studied a group of bacteria called hemoplasmas that live on the surface of red blood cells and can sometimes cause anemia. These bacteria are found in many animals, but very little is known about them in horses and donkeys. In Croatia, we tested 843 (817 horses and 26 donkeys) animals and found only one infected horse. The bacteria detected (*Mycoplasma wenyonii*) usually infect cattle, not horses. This shows that horses and donkeys are not natural hosts for these bacteria, but can sometimes become infected from other animals. Our study provides the first data of this kind from southeastern Europe.

## 1. Introduction

Hemotropic mycoplasmas (HM, hemoplasmas) are uncultivable, cell wall–less, epicellular bacteria that attach to the surface of red blood cells, potentially causing hemolytic anemia, reduced performance, and a range of non-specific systemic signs [1,2]. Based on molecular evidence in a wide range of hosts, these organisms are generally regarded as vector-borne pathogens transmitted by blood-sucking arthropods, although definitive proof of vector competence remains scarce. Transmission may also occur via direct blood contact or through iatrogenic means [2]. Furthermore, the possibility of intrauterine transmission cannot be excluded [3]. HMs are highly adapted to their hosts due to complex nutritional requirements and the ability to establish persistent infections [4].

While hemoplasmas are widely distributed among domestic and wild mammals, their occurrence in equids remains poorly understood. The first report of HM as a causative agent of disease in horses dates back to Nigeria in 1978 [5]. Infected animals may present with lethargy, inactivity, anorexia, weight loss, and pale mucous membranes due to varying degrees of anemia [5,6,7,8].

To date, no hemoplasma species has been identified as host-specific to equids. Limited studies have reported *Mycoplasma ovis* in horses from Iran and Brazil [9,10], closely related to *Candidatus* Mycoplasma haematobovis-like organisms and *Mycoplasma haemofelis* from Germany [6,7], *Mycoplasma haemocervae* from Nigeria [8], and *Candidatus* Mycoplasma haemovis as well as *Mycoplasma haemocanis* from Mexico [11], all detected by PCR and sequencing of the 16S rRNA gene.

Prevalence studies on HM in equids are extremely scarce. Only a few surveys worldwide—in Mexico, Brazil, Germany, Kyrgyzstan and Iran—have been conducted for molecular detection of hemoplasmas [7,9,10,11,12,13,14]. The findings of these studies are highly contrasting: two investigations in Brazil and Kyrgyzstan failed to detect hemoplasmas in any sampled animals, whereas others reported prevalences of 2.44% in Mexico, 4.55% in Brazil, 6.7% in Iran, and 26.5% in Germany. The lack of targeted surveillance and the absence of equid-specific species hinder understanding of their epidemiology, pathogenic potential, and transmission dynamics.

In the present study, we investigated the occurrence of HM in horses and donkeys from across Croatia, representing the first systematic survey of these pathogens in equids in the country. To our knowledge, no comparable studies have previously been conducted in southeastern Europe. This research therefore fills a critical gap in regional data, providing baseline information on the presence and molecular identity of hemoplasmas in equids from this part of the continent.

## 2. Materials and Methods

### 2.1. Study Area, Animals and Study Design

In our study, Croatian regions without sea access were classified as continental, while those with sea access were classified as coastal. A total of 549 horses were sampled from continental regions and 268 from coastal regions, while among 26 donkeys included, 20 originated from coastal and 6 from continental regions. Horses were stratified by age into four categories: foals (<1 year), young (1–6 years), adults (7–14 years), and seniors (≥15 years). Of the 817 horses examined, 2 were foals, 244 were young, 312 were adults, and 258 were seniors, with the youngest horse being 8 months old and the oldest 30 years. Donkeys were similarly grouped, with 13 classified as young, 11 as adults, and 2 as seniors, ranging in age from 2 to 24 years.

Animals were also categorized according to husbandry practices, reflecting the most common systems in Croatia. Horses from sport stables (*n* = 46) were housed in facilities with more than 20 animals kept exclusively for sport, with limited pasture access and consequently reduced exposure to vectors. Livery yards with more than 20 horses (*n* = 138) typically kept both sport and recreational horses, where grazing time was limited and herd size often delayed recognition of clinical signs. In smaller livery yards with up to 20 horses (*n* = 391), animals were mostly used for recreation and spent most of the day on pasture, resulting in increased vector exposure but easier recognition of disease signs. Horses kept at stables (*n* = 208) were under daily owner supervision, while those in free-roam systems (*n* = 34), predominantly cold-blooded horses, were left to graze freely with ruminants and rounded up in late autumn for health checks before being returned to pastures in spring. All donkeys were housed in stables, except six housed in livery yards with more than 20 animals.

The study included 67 stallions, 286 geldings, and 464 mares. Among 172 coldblooded horses, all but two belonged to indigenous Croatian breeds (the Croatian Coldblood horse and the Croatian Posavac), while the remaining 645 were warmblood horses of various breeds. In donkeys, 4 geldings, 6 stallions, and 15 jennies were sampled, all representing authentic Croatian breeds, namely the Littoral-Dinaric, Northern-Adriatic, and Istrian donkey. According to owner reports, 124 horses were used for sport, 135 for breeding, 41 were retired, and the rest were used for recreational riding, while all donkeys were kept for breeding purposes except for the four used recreationally.

All animals were examined for the presence of ectoparasites, including ticks, at the time of blood sampling.

The geographic origin was recorded for each animal. For better visibility of the obtained results, all locations were mapped using QGIS software version 3.30.0 RC.

### 2.2. Sample Collection and DNA Extraction

This sampling design ensured that animals of different ages, sexes, husbandry systems, and geographical origins were represented, providing a comprehensive overview of the equine population in Croatia. Blood samples were collected between 1 June 2024, and 31 July 2025. Sampling was performed in collaboration with local veterinarians and owners, ensuring representation of animals from various climatic zones, husbandry systems (pasture-based, mixed, and stable-kept), and purposes (recreational, breeding, and sport). Animals included both sexes and a wide range of age categories. Before sampling, the owners completed a questionnaire that included general information about the equids (age, breed, sex) as well as details about husbandry practices and the purpose of keeping the animals.

From each animal, approximately 5 mL of whole blood was obtained via jugular venipuncture using sterile, single-use needles and EDTA-coated vacutainer tubes. Samples were stored at 4 °C and transported to the Laboratory of Parasitology of Croatian Veterinary Institute within 48 h of collection. Upon arrival, each sample was aliquoted: one portion was used for direct microscopic examination (if applicable), and the remaining volume was stored at –20 °C until DNA extraction.

Genomic DNA was extracted from 200 μL EDTA blood samples using high-throughput settings with the KingFisher™ Flex automated instrument (Thermo Fisher Scientific, Waltham, MA, USA) and the MagMAX™ CORE Nucleic Acid Purification Kit (Applied Biosystems, Foster City, CA, USA).

### 2.3. Molecular Detection and Sequencing

Blood samples were screened by conventional PCR for *Mycoplasma* spp. using primers Myco322s and Myco938as targeting a 600 bp region of the 16S rRNA gene [15]. Furthermore, all samples were additionally screened using a PCR protocol targeting an approximately 800 bp fragment of the 23S rRNA gene, employing the primers 23S_HAEMO_F (5′-TGAGGGAAAGAGCCCAGAC-3′) and 23S_HAEMO_R (5′-GGACAGAATTTACCTGACAAGG-3′), as described by Mongruel et al. (2020) [16]. PCR reaction mixtures of 20 μL total volume were prepared, containing 10 μL G2 GOTaq master mix (Promega, Madison, WI, USA), 7.2 μL DNase/RNase-Free distilled water (Qiagen, Hilden, Germany), 0.4 μL of each 10 pmol/μL primer, and 2 μL of template DNA. The amplified products were analysed using capillary electrophoresis on the QIAxcel system (QIAGEN, Hilden, Germany).

For DNA sequencing, amplified PCR products were purified using the ExoSAP-IT-PCR Clean-Up Reagent according to the manufacturer’s instructions (USB Corporation, Cleveland, OH, USA). Sequencing of the amplified PCR product was performed by Macrogen Europe (Amsterdam, The Netherlands) using the same primers as for PCR. The sequences were assembled using the SeqMan Pro software 18, edited with EditSeq 18 in the Lasergene software package 18.0 (DNASTAR, Madison, WI, USA), and compared with available sequences using BLAST version 2.14.0.

## 3. Results

Out of a total of 817 horses and 26 donkeys examined across different regions of Croatia, only one horse tested positive for HM, corresponding to an overall prevalence of 0.12% (1/817; 95% CI: 0.003–0.68%). When extrapolated to the estimated national equine population of approximately 30,000 animals, this prevalence would translate into an estimated 36 potentially infected horses. However, given the wide confidence interval and the detection of a single case, this extrapolation should be interpreted with considerable caution.

**Figure 1:**
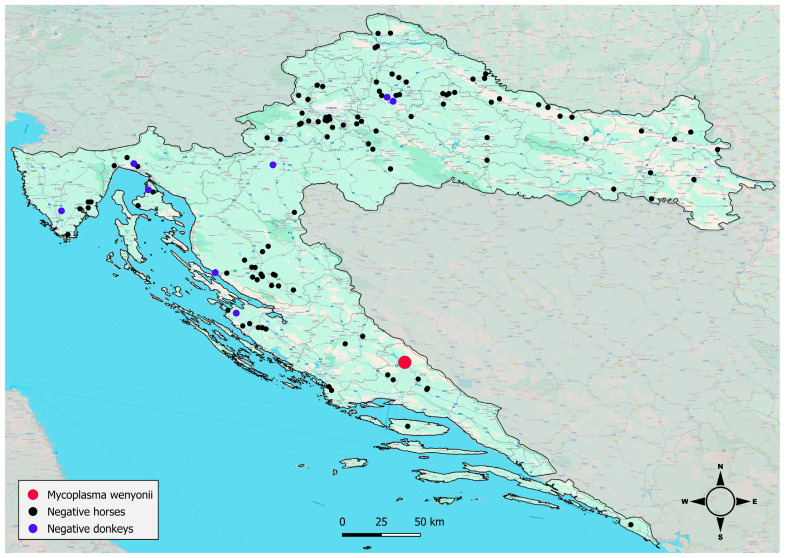
The map illustrates the sampling locations of horses and donkeys, providing an overview of the spatial distribution of samples and demonstrating coverage of all regions of Croatia. Dots represent sampling sites rather than individual animals.

The positive animal was an 11-year-old warmblood mare originating from a coastal region (Figure 1). The horse is housed in a livery yard with up to 20 horses, owned by her owner. She spends most days outside, grazing on pastures shared with cattle and other domestic ruminants. These grazing conditions increase the likelihood of interspecies contact and potential exposure to arthropod vectors. The infection was detected in July, while a blood sample collected from the same animal three months earlier, in April, had tested negative, suggesting that the infection was acquired in the intervening period. This temporal pattern points toward a recent transmission event, possibly linked to seasonal vector activity.

Despite the presence of *M. wenyonii* DNA in the blood sample, the mare did not exhibit any clinical signs of disease during repeated veterinary examinations. The animal remained in good general condition throughout the observation period. No ticks were detected in any of the infected mare.

Sequence analysis of a 585 bp fragment of the 16S rRNA gene confirmed that the amplified fragment showed 98.75% similarity, with 98% query coverage and an E-value of 0.0, to a *Mycoplasma wenyonii* sequence previously reported in aborted cattle fetuses from Croatia (GenBank accession number PV624262). An 831 bp fragment of the 23S rRNA gene was successfully sequenced. BLAST analysis revealed a 100% query coverage and an E-value of 0.0. The obtained sequence showed the highest similarity (99.28%) to *Mycoplasma wenyonii* sequences originating from the United States (GenBank accession numbers CP00373 and NR07692). High sequence similarity was also observed with several uncultured *Mycoplasma* spp., including 99.16% similarity to sequences detected in domestic pigs and blood-feeding *Culex* spp. from Thailand (GenBank accession number EP718825), 99.04% similarity to uncultured *Mycoplasma* spp. from domestic pigs and blood-feeding *Culex* spp. (GenBank accession numbers EP718826 and EP718829), and 98.92% similarity to uncultured *Mycoplasma* spp. identified in blood-feeding *Culex* spp. from Thailand (GenBank accession numbers EP718830 and EP718831). Additional similarities included 98.56% identity to an uncultured *Mycoplasma* sp. (GenBank accession number OM756781) and 98.44% identity to uncultured *Mycoplasma* spp. detected in blood-feeding *Culex* spp. and *Stomoxys calcitrans* from Thailand (GenBank accession numbers EP718827, EP7188328, and OM204159). The sequences were submitted to GenBank under the accession numbers 16S rRNA: PX832228 and 23S rRNA: PX797409.

## 4. Discussion

This study represents the first systematic investigation of HM in equids from southeastern Europe and, to our knowledge, only the second report of such infection in horses in Europe, following the cases described in Germany [6,7]. Our results revealed an exceptionally low prevalence of infection, with no evidence of host-specific hemoplasmas in either horses or donkeys, and only a single positive case involving *Mycoplasma wenyonii*, a species typically associated with ruminants. Given the large sample size of more than 800 horses from all regions of Croatia—collected across different seasons and including animals of various categories and uses—these results provide robust evidence for the apparent absence of equid-specific hemoplasmas in the studied population, but also in general as well. This is, in fact, the largest study of its kind to date, including more horses than all previous surveys combined. This raises an important question: why do equids, unlike many other mammalian hosts, lack their own host-adapted hemoplasma species?

Previous reports of hemoplasma-infected horses have mainly involved species typically associated with ruminants, such as *Mycoplasma ovis* [9,10], *Candidatus* Mycoplasma haematobovis-like [6,7], *Mycoplasma haemocervae* [8], and *Candidatus* Mycoplasma haemovis [11], as well as species more typical of carnivores such as *Mycoplasma haemofelis* (cats) or *M. haemocanis* (dogs). In our study, the single positive horse was infected with *M. wenyonii*, another hemoplasma species associated with cattle and previously detected in Croatia [3]. Although the route of infection remains unclear, the animal originated from a mountain region with extensive cattle grazing, suggesting possible transmission via shared pastures or hematophagous arthropods exposure. The animal spent most of the year grazing on communal pastures together with cattle and other ruminants. Since the infection was detected in July, the possibility of mechanical transmission by hematophagous insects should be considered. Interestingly, this horse had tested negative only a few months earlier, indicating that infection occurred relatively recently.

In contrast to successful PCRs detection, microscopic examination of blood smears did not reveal the presence of mycoplasmas. This discrepancy is most likely attributable to a low level of parasitemia, which is common in subclinical or chronic infections. These findings underscore the higher sensitivity of molecular methods for the detection of low-intensity infections and support the use of PCR as the method of choice for epidemiological studies and surveillance of HM.

Although the present finding represents a confirmed case of *M. wenyonii* in a horse, it may not necessarily be the first occurrence of this species in equids. Happi and Oluniyi (2020) [8] reported a sequence from a horse in Nigeria that shared 98.7% pairwise similarity with *M. ovis*, *Candidatus* M. haemocervae, and *M. wenyonii*. Interestingly, despite this high similarity, phylogenetic analysis grouped the sequence within the *Candidatus* M. haemocervae cluster. This is particularly intriguing, as *Candidatus* M. haemocervae has so far only been confirmed in sika deer (*Cervus nippon*) in Japan [17]. Furthermore, Byamukama et al. (2020) [18] documented both *M. ovis* and *M. wenyonii* in ruminants in Africa, suggesting that these hemoplasma species may have broader host ranges and geographic distributions than currently recognized. It is therefore plausible that the Nigerian horse was infected with either *M. ovis* or *M. wenyonii*, rather than *Candidatus* M. haemocervae.

These findings raise the possibility that cross-species transmission from ruminants to equids may occur, potentially facilitated by shared grazing areas, blood-sucking arthropods, or other mechanical routes of blood transfer. The extremely low prevalence observed in our study—may reflect the rarity of such spillover events and/or a short duration of detectable bacteremia in equids. While this hypothesis cannot be confirmed, it provides a biologically plausible explanation for the absence of host-specific hemoplasmas and the extremely low prevalence observed. In addition to the identification of non-equid hemotropic *Mycoplasma* species, the hypothesis that horses act as incidental hosts is further supported by findings from other studies in which HM were not detected in horses [13,14,19].

Although there is a paucity of data on ecological and epidemiological factors influencing hemoplasma infections in equids, evidence from other host–pathogen systems suggests that host specificity among HM is not absolute. While many hemoplasma species appear to be strongly adapted to a particular mammalian host group, sporadic detections in non host specific have been reported, indicating that cross-species transmission (“spillover”) can occur. For instance, *Mycoplasma ovis* has been documented in cervids [20], goats [21], sheep [16], and even humans [22]. Similarly, *M. wenyonii*, typically associated with cattle, has also been identified in sheep [23], cats [24] and *Bradypus tridactylus* [25].

In this context, the detection of *M. wenyonii* in a horse in our study provides additional evidence supporting the hypothesis that equids do not harbor their own host-specific hemoplasma species, but can occasionally acquire infections from other hosts. The transient nature of these spillover events, combined with potentially rapid clearance of the organism by the equine immune system, may explain both the extremely low prevalence observed and the absence of persistent hemoplasma species adapted to equids.

## 5. Conclusions

The current study represents the first systematic survey of HM in equids from southeastern Europe and only the second molecularly confirmed case in horses in Europe. Among 851 animals examined, only one horse tested positive for *M. wenyonii*, a ruminant-associated species, resulting in an overall prevalence of 0.12%. The absence of equid-specific hemoplasmas and the detection of a species typical of cattle support the view that equids are not natural reservoirs but may occasionally acquire infections through spillover. These findings underscore the need for further research on transmission routes, host specificity, and the epidemiological significance of hemoplasmas in equids.

## Data Availability

The datasets from the current study are available upon request to the corresponding author.

## References

[1] Messick J.B. (2004). Hemotrophic mycoplasmas (hemoplasmas): A review and new insights into pathogenic potential. Vet. Clin. Pathol..

[2] Arendt M., Stadler J., Ritzmann M., Ade J., Hoelzle K., Hoelzle L.E. (2004). Hemotrophic mycoplasmas-vector transmission in livestock. Microorganisms.

[3] Žilić D.J., Naletilić Š., Mihaljević Ž., Gagović E., Špičić S., Reil I., Duvnjak S., Tuk M.Z., Hodžić A., Beck R. (2025). Hemotropic pathogens in aborted fetuses of domestic ruminants: Transplacental transmission and implications for reproductive loss. Front. Microbiol..

[4] Hoelzle K., Ade J., Hoelzle L.E. (2020). Persistence in livestock mycoplasmas—A key role in infection and pathogenesis. Curr. Clin. Microbiol. Rep..

[5] Gretillat S. (1978). L’he’mobartonellose equine au Niger. Bull. Acad. Vet. Fr..

[6] Dieckmann S.M., Winkler M., Groebel K., Dieckmann M.P., Hofmann-Lehmann R., Hoelzle K., Wittenbrink M.M., Hoelzle L.E. (2010). Haemotrophic Mycoplasma infection in horses. Vet. Microbiol..

[7] Dieckmann S.M., Hoelzle K., Dieckmann M.P., Straube I., Hofmann-Lehmann R., Hoelzle L.E. (2012). Occurrence of hemotrophic mycoplasmas in horses with correlation to hematological findings. Vet. Microbiol..

[8] Happi A.N., Oluniyi P.E. (2020). A rare case of equine Haemotropic Mycoplasma infection in Nigeria. Niger. Vet. J..

[9] Kalantari M., Sharifiyazdi H., Ghane M., Nazifi S. (2020). The occurrence of hemotropic Mycoplasma ovis-like species in horses. Prev. Vet. Med..

[10] Kakimori M.T.A., Barros L.D., Collere F.C.M., Ferrari L.D.R., de Matos A., Lucas J.I., Coradi V.S., Mongruel A.C.B., Aguiar D.M., Machado R.Z. (2023). First molecular detection of Mycoplasma ovis in horses from Brazil. Acta. Trop..

[11] Ballados-González G.G., Cruz-Romero A., Martínez-Hernández J.M., Aguilar-Dominguez M., Vieira R.F.C., Grostieta E., Becker I., Sánchez-Montes S. (2025). Confirmation of the presence of Hemotropic Mycoplasma species in working equids from Veracruz, Mexico. Trop. Anim. Health Prod..

[12] Vieira R.F., Vidotto O., Vieira T.S., Guimaraes A.M., Santos A.P., Nascimento N.C., Santos N.J., Martins T.F., Labruna M.B., Marcondes M. (2015). Molecular investigation of hemotropic mycoplasmas in human beings, dogs and horses in a rural settlement in southern Brazil. Rev. Inst. Med. Trop. Sao Paulo.

[13] Valente J.D.M., Mongruel A.C.B., Machado C.A.L., Chiyo L., Leandro A.S., Britto A.S., Martins T.F., Barros-Filho I.R., Biondo A.W., Perotta J.H. (2019). Tick-borne pathogens in carthorses from Foz do Iguaçu City, Paraná State, southern Brazil: A tri-border area of Brazil, Paraguay and Argentina. Vet. Parasitol..

[14] Altay K., Erol U., Sahin O.F., Ulucesme M.C., Aytmirzakizi A., Aktas M. (2024). Survey of tick-borne pathogens in grazing horses in Kyrgyzstan: Phylogenetic analysis, genetic diversity, and prevalence of Theileria equi. Front. Vet. Sci..

[15] Varanat M., Maggi R.G., Linder K.E., Breitschwerdt E.B. (2011). Molecular prevalence of Bartonella, Babesia, and hemotropic Mycoplasma sp. in dogs with splenic disease. J. Vet. Intern. Med..

[16] Mongruel A.C.B., Spanhol V.C., Valente J.D.M., Porto P.P., Ogawa L., Otomura F.H., Marquez E.d.S., André M.R., Vieira T.S.W.J., Vieira R.F.d.C. (2020). Survey of vector-borne and nematode parasites involved in the etiology of anemic syndrome in sheep from Southern Brazil. Rev. Bras. Parasitol. Vet..

[17] Tagawa M., Matsumoto K., Yokoyama N., Inokuma H. (2013). Prevalence and Molecular Analyses of *Hemotrophic Mycoplasma* spp. (Hemoplasmas) Detected in Sika Deer (Cervus nipponyesoensis) in Japan. J. Vet. Med. Sci..

[18] Byamukama B., Tumwebaze M.A., Tayebwa D.S., Byaruhanga J., Angwe M.K., Li J., Galon E.M., Liu M., Li Y., Ji S. (2020). First Molecular Detection and Characterization of Hemotropic Mycoplasma Species in Cattle and Goats from Uganda. Animals.

[19] Ferreira E.P., Vidotto O., Almeida J.C., Ribeiro L.P., Borges M.V., Pequeno W.H., Stipp D.T., de Oliveira C.J., Biondo A.W., Vieira T.S. (2016). Serological and molecular detection of Theileria equi in sport horses of northeastern Brazil. Comp. Immunol. Microbiol. Infect. Dis..

[20] André M.R., Duarte J.M.B., Gonçalves L.R., Sacchi A.B.V., Jusi M.M.G., Machado R.Z. (2020). New records and genetic diversity of Mycoplasma ovis in free-ranging deer in Brazil. Epidemiol. Infect..

[21] Machado C.A.L., Vidotto O., Conrado F.O., Santos N.J.R., Valente J.D.M., Barbosa I.C., Trindade P.W.S., Garcia J.L., Biondo A.W., Vieira T.S.W.J. (2017). Mycoplasma ovis infection in goat farms from northeastern Brazil. Comp. Immunol. Microbiol. Infect. Dis..

[22] Maggi R.G., Compton S.M., Trull C.L., Mascarelli P.E., Mozayeni B.R., Breitschwerdt E.B. (2013). Infection with hemotropic Mycoplasma species in patients with or without extensive arthropod or animal contact. J. Clin. Microbiol..

[23] Murugesan A.C., Kumaragurubaran K., Gunasekaran K., Murugasamy S.A., Arunachalam S., Annamalai R., Ragothaman V., Ramaswamy S. (2024). Molecular Detection of Hemoplasma in animals in Tamil Nadu, India and Hemoplasma genome analysis. Vet. Res. Commun..

[24] Álvarez-Fernández A., Maggi R., Martín-Valls G.E., Baxarias M., Breitschwerdt E.B., Solano-Gallego L. (2022). Prospective serological and molecular cross-sectional study focusing on Bartonella and other blood-borne organisms in cats from Catalonia (Spain). Parasit. Vectors.

[25] de Oliveira L.B., Calchi A.C., Vultão J.G., Yogui D.R., Kluyber D., Alves M.H., Desbiez A.L.J., de Santi M., Soares A.G., Soares J.F. (2022). Molecular investigation of haemotropic Mycoplasmas and Coxiella burnetii in free-living Xenarthra mammals from Brazil, with evidence of new haemoplasma species. Transbound. Emerg. Dis..

